# Prediction of Thermomechanical Behavior of Wood–Plastic Composites Using Machine Learning Models: Emphasis on Extreme Learning Machine

**DOI:** 10.3390/polym17131852

**Published:** 2025-07-02

**Authors:** Xueshan Hua, Yan Cao, Baoyu Liu, Xiaohui Yang, Hailong Xu, Lifen Li, Jing Wu

**Affiliations:** 1 Special and Key Laboratory for Development and Utilization of Guizhou Superior Bio-Based Materials, Guizhou Minzu University, Guiyang 550025, China; 20230714000029@stu.gzmu.edu.cn (X.H.); 201301030@gzmu.edu.cn (Y.C.); liubaoyu1020@163.com (B.L.); xiaohuiyang90@163.com (X.Y.); 2 College of Data Science and Information Engineering, Guizhou Minzu University, Guiyang 550025, China; 3 Engineering Research Center of Green and Low-Carbon Technology for Plastic Application, Guizhou Minzu University, Guiyang 550025, China; 4 College of Forestry, Guizhou University, Guiyang 550025, China

**Keywords:** wood–plastic composites, dynamic thermomechanical properties, loss modulus, machine learning, extreme learning machine

## Abstract

The dynamic thermomechanical properties of wood–plastic composites (WPCs) are influenced by various factors, such as the selection of raw materials and processing parameters. To investigate the effects of different wood fiber content ratios and temperature on the loss modulus of WPCs, seven different proportions of Masson pine (*Pinus massoniana* Lamb.) and Chinese fir [*Cunninghamia lanceolata* (Lamb.) Hook.] mixed-fiber-reinforced HDPE composites were prepared using the extrusion molding method. Their dynamic thermomechanical properties were tested and analyzed. The storage modulus of WPCs showed a decreasing trend with increasing temperature. A reduction in the mass ratio of Masson pine wood fibers to Chinese fir wood fibers resulted in an increase in the storage modulus of WPCs. The highest storage modulus was achieved when the mass ratio of Masson pine wood fibers to Chinese fir wood fibers was 1:5. In addition, the loss modulus of the composites increased as the content of Masson pine fiber decreased, with the lowest loss modulus observed in HDPE composites reinforced with Masson pine wood fibers. The loss tangent for all seven types of WPCs increased with rising temperatures, with the maximum loss tangent observed in WPCs reinforced with Masson pine wood fibers and HDPE. A prediction method based on the Extreme Learning Machine (ELM) model was introduced to predict the dynamic thermomechanical properties of WPCs. The prediction accuracy of the ELM model was compared comprehensively with that of other models, including Support Vector Machines (SVMs), Random Forest (RF), Back Propagation (BP) neural networks, and Particle Swarm Optimization-BP (PSO-BP) neural network models. Among these, the ELM model showed superior data fitting and prediction accuracy, with an *R*^2^ value of 0.992, Mean Absolute Error (MAE) of 1.363, and Root Mean Square Error (RMSE) of 3.311. Compared to the other models, the ELM model demonstrated the best performance. This study provides a solid basis and reference for future research on the dynamic thermomechanical properties of WPCs.

## 1. Introduction

Wood–plastic composites (WPCs) are innovative materials with high strength, waterproof, corrosion resistance, insect resistance, and energy-saving and environmentally friendly properties. They combine the benefits of both wood and plastic, making them suitable for a wide range of applications, including landscaping [1,2], building [3,4], automotive interiors decoration [5], and furniture manufacturing [6].

The dynamic thermomechanical properties of WPCs are influenced by various factors, including the selection of reinforcing materials, component ratios, preparation methods, and processing parameters. Koffi et al. [7] found that both the storage modulus and loss modulus of WPCs increase as the fiber ratio increases. Mazzanti et al. [8] observed similar trends. Nurazzi et al. [9] discovered that alkaline treatment of sugar palm fibers enhances the storage modulus of WPCs. Gupta et al. [10] analyzed the thermomechanical properties of hybrid composites reinforced with jute and sisal fibers. In these composites, the total fiber content was maintained at 30% of the total weight. The results showed that WPCs with a higher sisal fiber content (jute to sisal ratio of 25:75) exhibited a higher storage modulus and loss modulus compared to those with 50:50 and 75:25 ratios. To optimize the performance and expand their range of applications, further understanding of the dynamic thermomechanical properties and behavior of WPCs is essential. However, current research on the mechanical properties of WPCs still largely relies on traditional experimental methods, which are time-consuming and labor-intensive.

Machine learning is an interdisciplinary field that encompasses knowledge from computer science, probability theory, and statistics [11,12]. The fundamental principle of machine learning involves using algorithms to extract features from large datasets, building predictive models, and applying these models to classify and predict new data. Machine learning has become one of the most promising research methods in material property prediction and new material screening, owing to its strong generalization ability and fast computation speed [13,14]. Several scholars have applied machine learning algorithms to predict the mechanical properties of composites. For example, Zhang et al. [15] constructed a Back Propagation (BP) neural network model to predict the mechanical properties of stainless steel ultra-thin strips after heat treatment. Wang et al. [16] developed models using Random Forest (RF), Support Vector Machines (SVMs), BP neural network, and genetic-algorithm-optimized BP neural network models to predict the elastic modulus of titanium alloys. Additionally, Wang et al. [17] used four machine learning algorithms—multiple linear regression, BP neural network, RF, and XGBoost—to establish a predictive model for the mechanical properties of steel strips. These studies indicate that machine learning has great potential for development in the field of composite mechanical property prediction.

Currently, the use of machine learning algorithms to predict the mechanical properties of WPCs is still in the exploratory and developmental stage, with limited available references. Sun et al. [18] used both a BP neural network and a BP neural network optimized by a genetic algorithm to predict internal bonding strength, static bending strength, and water absorption thickness swelling rate. The prediction errors for the BP neural network model ranged from 8% to 1491%, 2.8% to 1950%, and 15% to 128%, respectively. In contrast, the prediction error ranges of the PSO-BP neural network model were 2% to 15.5%, 9% to 38%, and 4% to 70%, respectively, indicating a significant improvement in prediction accuracy. However, the lack of data preprocessing may have affected the accuracy of the model due to the influence of different data dimensions. Cai et al. [19] selected an SVM model with a Gaussian radial basis function (GRBF) kernel to predict the tensile strength and impact toughness of straw-reinforced composites, using a BP neural network as a comparative model. The Mean Relative Errors (MREs) for the SVM model in predicting tensile strength and impact toughness were 2.12% and 4.89%, respectively, while the BP neural network model had MREs of 5.43% and 8.86%, respectively. The MREs of the SVM model were significantly lower than those of the BP neural network. However, the analysis of the regression capabilities of the models only used the MRE as the evaluation metric, which is a relatively single criterion and fails to provide a comprehensive assessment of the predictive performance of the models. Qin et al. [20] combined a grey neural network with a BP neural network to predict the shear modulus of WPCs. The experimental results showed that this combined model outperformed other single models in terms of accuracy.

Chen et al. [21] used four models, including ridge regression, Lasso, Multilayer Perceptron (MLP), and Elastic Net, to predict the tensile strength, bending strength, and bending modulus of WPCs. Among these models, the MLP had the highest prediction accuracy, with values of 94.2%, 90.0%, and 92.5%, respectively, while all four models captured the relationship between the original data based on a small amount of sample data and predicted the mechanical properties of WPCs with reasonable accuracy. However, the generalization ability of these models has not been thoroughly validated, and there is still potential for further optimization to improve the prediction accuracy. The Extreme Learning Machine (ELM) is a widely used learning algorithm and has been extensively applied in predicting various properties of polymers, such as mechanical behavior, thermal weight performance, thermal conductivity, refractive index, electrical conductivity, inherent viscosity, ionic conductivity, and refractive index [22], etc. Despite its promising potential, machine learning in material property predicting faces challenges such as poor generalization ability and overfitting in practical applications. In the case of predicting the mechanical properties of WPCs, data acquisition is costly and time-consuming, and the dataset is usually small. A small-sample dataset leads to insufficient model training, making it difficult for the model to accurately capture the relationship between material properties and various influencing factors, and the model is prone to overfitting. Meanwhile, training models using only a single dataset results in poor generalization. To overcome these limitations, techniques like cross validation or regularization methods can be used.

Based on these above, in this study, seven different ratios of Masson pine (*Pinus massoniana* Lamb.) and Chinese fir [*Cunninghamia lanceolata* (Lamb.) Hook.] mixed-fiber-reinforced HDPE composites were prepared using the extrusion molding method. The dynamic thermomechanical properties of these composites were tested and analyzed. Predictions were made using the ELM model, and the results were compared with models such as RF, SVM, and BP neural network. This research provides new insights and methods for predicting the mechanical properties of WPCs.

## 2. Materials and Methods

### 2.1. Material Preparation

Chinese fir fibers and Masson pine fibers with lengths ranging from 0.18 to 0.85 mm were placed in a forced-air-drying oven and dried at 105 °C for 24 h until the moisture content was lower than 3%. The wood fibers, HDPE (5000S), and MAPE (GMG 9804), were thoroughly mixed in a high-speed mixer for over 20 min, according to the proportion shown in Table 1. HDPE was provided by Daqing Petrochemical Company, China, with a solid density of 954 kg/m^3^, a melting flow index of 0.35 g/10 min (measured at 230 °C and 2.16 kg according to ISO 1133), a flexural strength of 24 MPa, and a tensile strength of 29 MPa. MAPE was produced by Tianjin Bochen Co., Ltd., Tianjin, China.

Composite strips reinforced with Chinese fir fibers and Masson pine fibers with a cross-sectional size of 40 mm × 4 mm were prepared using a two-step process of compounding and extrusion. The speed of the twin-screw extruder (SJSH 30 mm) was set at 40 rpm, and that of the single-screw extruder (SJ 45 mm) at 15 rpm. The temperature was maintained between 150 and 175 °C during lumber extrusion. A total of seven different types of composite strips were produced.

### 2.2. Dynamic Thermomechanical Property Testing and Analysis Method

The dynamic thermomechanical properties of seven HDPE composites, reinforced with a blend of Chinese fir and Masson pine fibers, were evaluated using a three-point bending test. Testing was conducted over a temperature range of 20 to 120 °C, with a heating rate of 5 °C/min and a frequency of 1 Hz. Each specimen measured 40 mm in length, 10 mm in width, and 3 mm in thickness. Three specimens were tested for each group of WPCs.

### 2.3. The Support Vector Machine Model

The SVM is a supervised learning algorithm suitable for handling small sample sizes, nonlinear data, and high-dimensional data. It is primarily used to solve classification and regression problems [23]. The core idea of an SVM is to classify data points by finding the optimal hyperplane in the feature space, which maximizes the margin between different classes. When dealing with classification problems, an SVM maps the data into a high-dimensional space and finds the optimal hyperplane in this space, thereby enabling better linear separation of different sample points in the new feature space. Assuming a given sample set $D=\left\{\left(x_{1},y_{1}),(x_{2},y_{2}),\cdots ,(x_{n},y_{n}\right)\right\}$, mapping it to a high-dimensional space to construct a linear function is:

(1) f(x)=ω⋅x+b

where *ω* is a weight vector; *b* is the bias term; and *x* represents the input feature vector.

Support Vector Regression (SVR) is a regression algorithm based on SVM, primarily used to solve regression problems. Like an SVM, SVR maps the data into a high-dimensional space. However, instead of finding the optimal hyperplane for classification, SVR performs linear regression fitting in this high-dimensional space to find the optimal hyperplane that best fits the data points. This hyperplane is typically represented as a regression function. SVR has several advantages, including strong generalization ability, convergence to a global optimum, and insensitivity to the dimensionality of the input data. The prediction performance of the SVR model is significantly influenced by its kernel function, and the choice of different kernel functions can lead to varying levels of prediction accuracy. Commonly kernel functions used In SVR are listed in Table 2. The ultimate optimization objective function for SVR is:

(2) $$f{\left(x\right)}_{SVR}=\frac{1}{2}{\left\|\omega \right\|}^{2}+C\sum_{i=1}^{n}\left(\xi_{i}+\xi_{i}^{*}\right)$$

where *C* is the regularization parameter; and $\xi_{i}$ and $\xi_{i}^{*}$ are relaxation variables.

### 2.4. The Extreme Learning Machine Model

**Figure 1:**
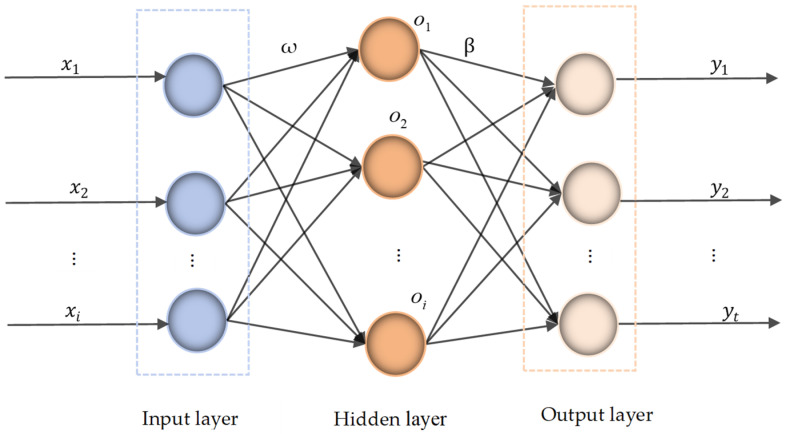
The ELM network structure diagram.

ELM is a single-hidden-layer feed-forward neural network [24]. Its network structure is shown in Figure 1. In ELM, the number of neurons in the hidden layer is determined, and the input weights *ω* and biases *b* are randomly generated. The hidden layer output matrix *H* is calculated using an activation function, and the output layer weights *β* = *HT* are then computed. Here, *β* represents the output weights, and *T* is the expected output. Compared to traditional BP neural networks, the ELM algorithm has several advantages, including fewer training parameters, higher learning efficiency, and stronger generalization ability. Both the ELM and SVM models have similar structures, and the ELM model is also suitable for processing small-sample data.

### 2.5. The Particle Swarm Optimization Algorithm

PSO is an intelligent optimization algorithm that simulates the group behavior of birds, fish, and other social organisms to find the optimal solution. In the PSO algorithm, each particle adjusts its velocity and direction based on its own best position and the global best position. Through continuous iteration, the particles update their positions and velocities, eventually converging to the global optimal solution or an approximate optimal solution.

The position of the i particle is $X_{id}=\left(x_{i1},x_{i2},\cdots ,x_{iD}\right)$.

The speed of the i particle is $V_{id}=\left(v_{i1},v_{i2},\cdots ,v_{iD}\right)$.

The optimal position found by the i particle is $P_{id,pbest}=\left(p_{i1},p_{i2},\cdots ,p_{iD}\right)$.

The optimal position found by the group search is $P_{d,gbest}=\left(p_{1,gbest},p_{2,gbest},\cdots ,p_{D,gbest}\right)$.

The formula for updating the position and velocity of population particles is as follows:

(3) $$v_{id}^{k+1}=\omega v_{id}^{k}+c_{1}r_{1}\left[p_{id,pbest}^{k}-x_{id}^{k}\right]+c_{2}r_{2}\left[p_{d,gbest}^{k}-x_{id}^{k}\right]$$

(4) $$x_{id}^{k+1}=x_{id}^{k}+v_{id}^{k+1}$$

where ω is the inertia weight, $c_{1}$ and $c_{2}$ are learning factors, k is the number of iterations, $r_{1}$ and $r_{2}$ are random numbers, with a range of [0, 1], $p_{id,pbest}^{k}$ is the optimal solution found by the i particle after the k iteration, $p_{d,gbest}^{k}$ is the optimal solution of the entire particle swarm after the k iteration, $v_{id}^{k}$ is the velocity vector of particle i in the d dimension at the k iteration, and $x_{id}^{k}$ is the position vector of particle i in the d dimension at the k iteration.

### 2.6. The Random Forest Model

RF is an ensemble learning method that integrates multiple weak learners to form a strong learner, processing classification or regression prediction problems by combining multiple decision trees [25]. RF can effectively handle high-dimensional datasets and has robustness to missing values and outliers in the features. By introducing randomness into the model, RF reduces its dependence on the training data, thus minimizing the risk of overfitting. While less interpretable than a single decision tree, RF generally offers better generalization.

### 2.7. The PSO-BP Neural Network Model

**Figure 2:**
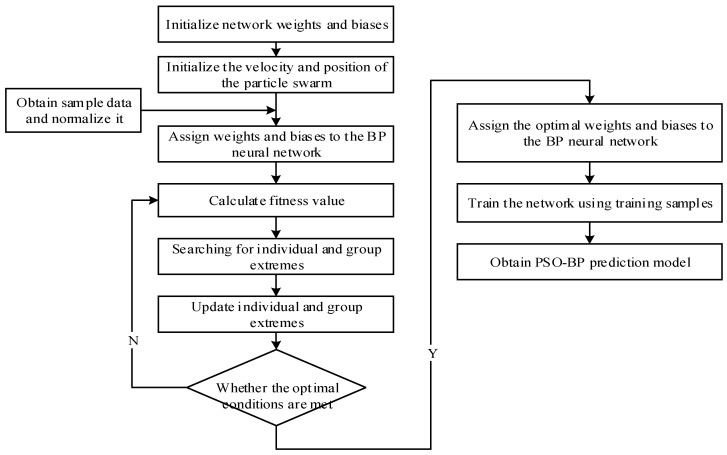
Flowchart of the PSO-BP algorithm.

The BP neural network is a multi-layer feedforward neural network that is trained using the error backpropagation algorithm. It is widely utilized in predicting material mechanics performance. The BP neural network consists of an input layer, one or more hidden layers, and an output layer, each containing multiple neurons. The input data is transmitted through the neurons and processed via an activation function to produce output values. The error between these output values and the true values is calculated, and through BP, the weights and biases of each neuron are adjusted to minimize the error until the predetermined training target is achieved. However, the BP neural network often suffers from issues such as slow convergence and a tendency to become stuck in local optima. To address these problems, the PSO algorithm is introduced to optimize the initial weights and biases of the neural network. This approach reduces the likelihood of falling into local optima and improves the convergence speed and prediction accuracy of the network. The algorithm flow is shown in Figure 2.

### 2.8. Model Evaluation

K-fold cross validation is a method that randomly divides the original dataset into K mutually exclusive subsets. In each iteration, one subset is selected as the test set and the remaining K-1 subsets as used for training. The process is repeated K times so that each subset participates in the training and testing of the model. The mean and standard deviation of the K test results are calculated as the final evaluation of the model’s generalization performance. This method is particularly useful for small-sample datasets.

In regression analysis studies, the commonly used evaluation indicators include *R*^2^, MSE, RMSE, MAE, MRE, and *r*. *R*^2^ reflects the ability of model to explain the variation in the dependent variable. It ranges from 0 to 1, with a value closer to 1 indicating a better fit. The MSE represents the mean squared error, and the RMSE is the square root of the MSE. The value of the MSE is more difficult to interpret than the RMSE; so, the RMSE is usually used to evaluate the model. The MAE is known as the absolute loss, which indicates how close the predicted value is to the actual value; the smaller the MAE is, the closer the predicted value is to the actual value. The MRE measures the proportion of the prediction error relative to the actual value and is used to standardize the error; the smaller the MRE is, the smaller the relative prediction error of the model is. The correlation coefficient, r, measures the linear relationship between two variables.

To better evaluate the prediction performance of the model, five evaluation metrics, including *R*^2^, *r*, MRE, MAE, and RMSE, were chosen as the criteria for assessing the model. The calculation formulas for these metrics are as follows:

(5) $$R^{2}=1-\frac{{\sum_{i=1}^{n}\left(y_{i}-{\hat{y}}_{i}\right)}^{2}}{{\sum_{i=1}^{n}\left(y_{i}-{\bar{y}}_{i}\right)}^{2}}$$

(6) $$r=\rho \left(y_{i},{\hat{y}}_{i}\right)=\frac{cov\left(y_{i},{\hat{y}}_{i}\right)}{\sigma_{y_{i}}\sigma_{{\hat{y}}_{i}}}$$

(7) $$\mathrm{MRE}=\frac{1}{n}\sum_{i=1}^{n}\left|\frac{y_{i}-{\hat{y}}_{i}}{y_{i}}\right|$$

(8) $$\mathrm{MAE}=\frac{1}{n}\sum_{i=1}^{n}\left|y_{i}-{\hat{y}}_{i}\right|$$

(9) $$\mathrm{RMSE}=\sqrt{\frac{1}{n}{\sum_{i=1}^{n}\left(y_{i}-{\hat{y}}_{i}\right)}^{2}}$$

where n is the number of samples, $y_{i}$ and ${\hat{y}}_{i}$ are the experimental and predicted values of the i sample, respectively, $\bar{y}$ is the average of the experimental value, $\mathrm{cov}(y_{i},\;{\hat{y}}_{i})$ is the covariance between $y_{i}$ and ${\hat{y}}_{i}$, and $\sigma_{y_{i}}$ and $\sigma_{{\hat{y}}_{i}}$ are the standard deviations of $y_{i}$ and ${\hat{y}}_{i}$, respectively.

## 3. Machine Model Establishment

A prediction model for the mechanical properties of the loss modulus of WPCs has been established, incorporating input variables such as Masson pine fiber content, Chinese fir fiber content, and temperature. The modeling techniques employed include an SVM with different kernel functions, RF, ELM, BP neural network models, and PSO-BP neural network models. The model construction process is as follows:

(1) Data preprocessing: Prior to model training, the sample data must be preprocessed. Among them, normalization is an important step, usually normalizing sample data to the range of [0, 1]. Normalization ensures that all features have the same range of values, prevents certain features from affecting the model due to a large numerical range, accelerates the convergence speed, and improves the generalization ability of the model. After the training is completed, in order to better analyze the prediction performance of the model, it is necessary to denormalize the data and return to the range of the original data. In this paper, the Min–Max normalization method is used to normalize the data in the range of [0, 1]. The specific formula is shown in Equation (10), where *X* is the raw data value to be processed, and *X*_max_ and *X*_min_ are the maximum and minimum values of the variable, respectively.

(10) $$Y=\frac{X-X_{\min}}{X_{\max}-X_{\min}}$$

(2) Model training: The SVR-Linear, SVR-Poly, SVR-RBF, RF, BP, PSO-BP, ELM models are built separately, and the models are validated using five-fold cross validation.
(3) Model performance evaluation: To evaluate performance, K-5-fold cross validation is implemented by shuffling the 147 sample data points. The dataset is randomly divided into five subsets; four subsets are used for training in each iteration, while the remaining subset is employed for testing. This process is repeated five times to obtain five sets of predicted values, with the final predicted value calculated as the mean of these five sets.
(4) Model parameter settings: The BP neural network has a three-layer network structure. The PSO algorithm is introduced into the BP model to optimize the initialization of weights and biases. The optimization range is [0, 1], and the weight from the hidden layer to the output layer after optimization is 0.112; the bias of the output layer is 0.405. The parameter settings of the model are shown in Table 3.

## 4. Results and Discussion

### 4.1. The Storage Modulus of Wood–Plastic Composites Varies with Temperature

The storage modulus refers to the ability of a materials to store and release energy when subjected to external force, which is an important indicator of elasticity of material.

**Figure 3:**
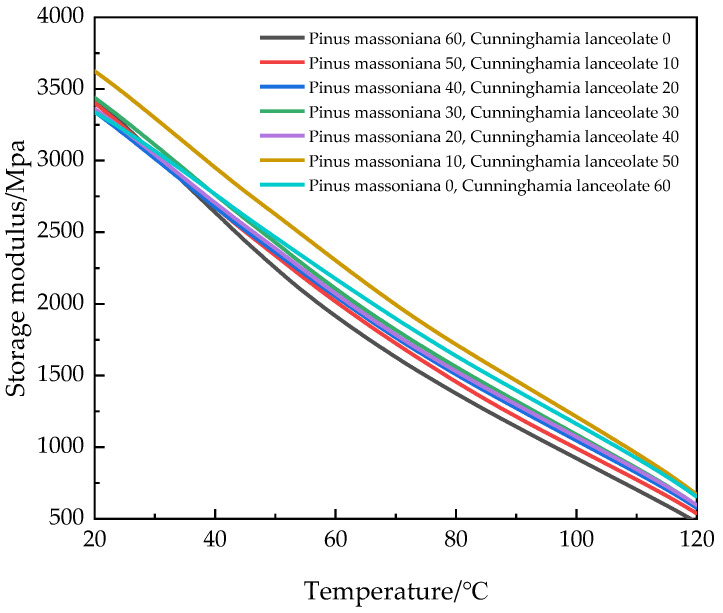
Relationship between storage modulus and temperature of WPCs.

Figure 3 shows the temperature dependence curves of the storage modulus of the seven groups of HDPE composites reinforced with mixed fibers of Masson pine wood and Chinese fir wood. As the temperature increased, the molecular chain within the seven groups of WPCs became more active, leading to a decrease in elasticity and a downward trend in the storage modulus [26]. Notably, as the mass ratio of Masson pine wood fibers to Chinese fir wood fibers decreased, the storage modulus of WPCs showed an upward trend. When the mass ratio of Masson pine wood fibers to Chinese fir wood fibers was 1:5, the storage modulus of the WPCs reached the highest value. This improvement is attributed to the increased content of Chinese fir wood fiber, which enhances interfacial compatibility between the fibers and the matrix, thereby improving the elastic modulus and hardness of the WPCs.

### 4.2. The Loss Modulus of Wood–Plastic Composites Varies with Temperature

The loss modulus refers to the ability of materials to dissipate energy or convert it to heat during deformation, serving as a measure of energy loss in materials.

**Figure 4:**
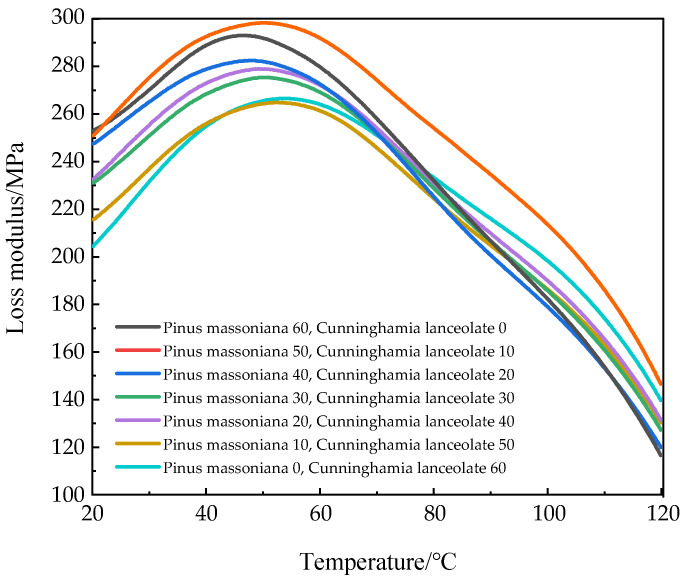
Relationship between loss modulus and temperature of WPCs.

Figure 4 shows the relationship between the loss modulus of the seven HDPE composites reinforced with Masson pine wood fibers and Chinese fir wood fibers and temperature. The loss modulus of the composites increased with the decrease in the content of Masson pine fiber, and the lowest value of loss modulus was found in the HDPE composites reinforced with Masson pine wood fiber. When the temperature approached 50 °C, the WPCs exhibited successive relaxation transition peaks. The smaller the mass ratio of Masson pine wood fibers to Chinese fir wood fibers, the less energy was required for the composites to melt and flow [27].

### 4.3. The Loss Tangent of Wood–Plastic Composites Varies with Temperature

**Figure 5:**
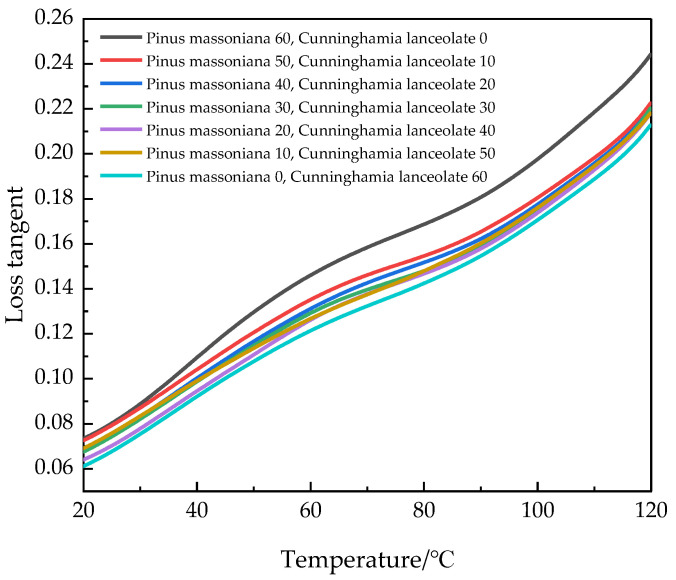
Relationship between loss tangent and temperature of WPCs.

The loss tangent reflects the damping characteristics and viscoelastic properties of the material. As shown in Figure 5, the loss tangent of all seven types of WPCs increases with rising temperature. This increase occurs because higher temperatures amplify the movement of the HDPE molecular chains, leading to greater frictional losses between them.

The highest loss tangent was observed in the WPCs reinforced with Masson pine wood fibers and HDPE, indicating that the rigidity of Masson pine wood fibers was lower than that of Chinese fir wood fibers, while its toughness was higher. The smallest increase in loss tangent with temperature rise was seen in WPCs reinforced with Chinese fir wood fibers and HDPE, where energy loss was minimal. This further demonstrated the strong interface bonding performance between Chinese fir wood fibers and HDPE.

### 4.4. The SVR Model

**Figure 6:**
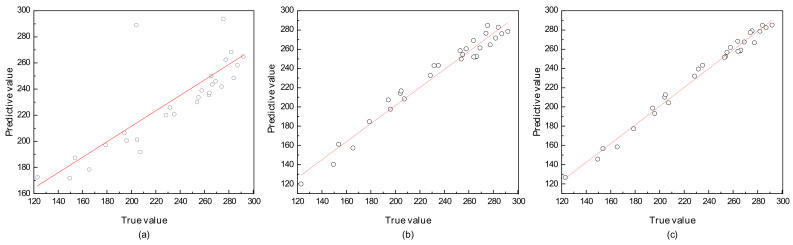
Prediction results of the different kernel function models on the test set. (**a**) SVR-Linear, (**b**) SVR-Poly, (**c**) SVR-RBF.

To investigate the impact of different kernel functions on the predictive accuracy of the model, three kernel functions, namely, Linear kernel function, Polynomial kernel function, and the Gaussian radial basis kernel function, were selected to build the models. The prediction results of the three models on the test set are shown in Figure 6, where the dashed line represents the regression line when the predicted values equal the true values, and the scattered points represent the predicted values. The experimental results indicate that the predictive accuracy of the Gaussian radial basis and Polynomial kernel functions was higher than that of the linear kernel function.

The comparison of error indicators for various kernel function models on the test set is shown in Table 4. The *R*^2^ values for the SVR-Linear, SVR-Poly, and SVR-RBF models were 0.547, 0.960 and 0.985, respectively. The *R*^2^ value of the SVR-RBF model was higher than those of the SVR-Linear and SVR-Poly models, indicating that the SVR-RBF model demonstrated superior data-fitting ability. The MAE, MRE, and RMSE values for the SVR-RBF model were 4.452, 0.021 and 5.296, respectively, all of which were significantly lower than those of the SVR-Linear and SVR-Poly models. This indicated that the predictive accuracy of the GRBF kernel is higher than that of the other two models.

The experimental results indicate that the GRBF kernel had high data-fitting ability and predictive accuracy in regression prediction. This provided an important reference for the selection of kernel functions in SVM when dealing with regression prediction problems. However, the choice should still be comprehensively considered based on the characteristics of different problems and datasets.

### 4.5. The ELM Model

**Figure 7:**
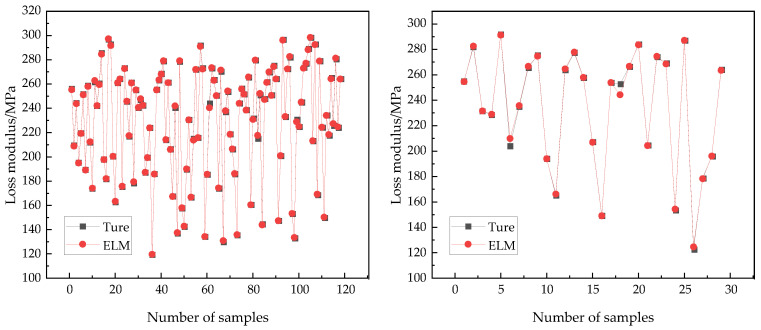
Comparison of prediction performance between training set (**left**) and testing set (**right**).

Figure 7 illustrates the prediction performance of the ELM model on both the training set and test set. The higher the degree of overlap between the two curves, the greater the predictive accuracy of the ELM model. Combined with Table 5, it can be seen that the *R*^2^ values of the ELM model on both the training set and test set exceed 0.9, indicating that the ELM model had strong data-fitting and generalization capabilities, with a small difference between the predicted and actual values. In material performance prediction, the RMSE quantifies the closeness between the predicted values of the model and the true values. The smaller the value, the more accurate the model’s prediction of the material. The MAE is the average absolute difference between the predicted and actual values, providing an intuitive measure of the model’s average prediction error. The MAE can help researchers evaluate the impact of prediction errors on the actual properties and applications of materials from the perspective of average error, and determine whether model improvements are needed.

The MAE values of the ELM model on the training and test set were 0.351 and 1.363, respectively, indicating a decrease in the prediction accuracy on new datasets. The MRE values of model were 0.003 and 0.526, respectively, showing a significant increase. The RMSE values were 0.504 and 3.311, respectively, indicating a decline in prediction performance on the test set. Although the *R*^2^ value of the ELM model was very high, the increased MAE, MRE, and RMSE values on the test set suggested that the model showed signs of overfitting.

### 4.6. The Random Forest Model

**Figure 8:**
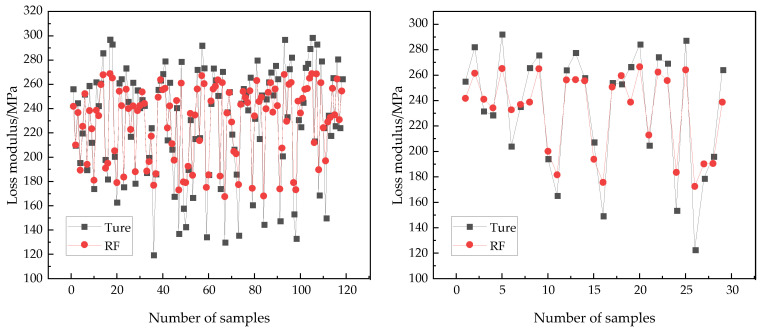
Comparison of prediction performance of training set (**left**) and testing set (**right**).

The prediction results of the RF model on the training and testing sets are shown in Figure 8. Overall, the RF model effectively captures the trend of changes in the true values; however, there is still some deviation between the predicted and actual values, indicating that the prediction accuracy of the model needs further improvement. Table 6 presents a comparison of the evaluation results for the RF model on the training and test set. The *R*^2^ values of the RF model on the training and test set were 0.847 and 0.799, respectively, suggesting that the *R*^2^ value on the training set was significantly higher than that on the test set, which suggests that the RF model has a higher degree of fit on the training set and a lower degree of fit on the new dataset. The MAE values for the RF model were 13.705 for the training set and 15.864 for the test set, while the RMSE values were 17.777 and 19.849, respectively. The MRE values were 6.831 for the training set and 7.914 for the test set. These results indicate that the RF model performs better on the training set than on the test set, indicating that the RF model suffers from overfitting and poor generalization ability on new datasets. In RF, the number of decision trees is 500, and the model has a large number of trees. The model is too complex, and the small number of training samples leads to overfitting.

### 4.7. The BP and PSO-BP Model

**Figure 9:**
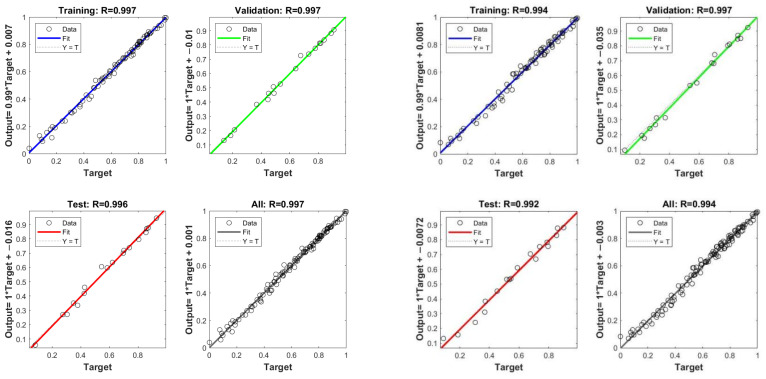
Regression ability analysis of BP neural network model (**left**); PSO-BP neural network model (**right**).

Figure 9 presents the prediction results of the BP and PSO-BP neural network models. The scatter points represent the normalized values of the sample data, the solid lines represent the best fit line, and the dashed lines indicate the fit line when the actual values match the predicted values.

The values of *r* for the BP and PSO-BP neural network models on the training subset, test subset, validation subset, and the entire training set were 0.997, 0.997, 0.996, 0.997 and 0.994, 0.997, 0.992, 0.994, respectively. The slight increase in the value of *r* for the PSO-BP model indicates that the PSO algorithm can effectively optimize the weights and biases of the BP neural network model.

According to Table 7, the PSO-BP model outperforms the BP neural network in the evaluation metrics of *R*^2^, MAE, and RMSE on both training and testing sets, with an improvement of 0.893%, 31.19%, and 25.33%, respectively. This indicates that the PSO-BP model has a stronger prediction ability, and the transformed BP neural network model has a higher data-fitting ability and prediction accuracy. The introduction of the Particle Swarm Optimization Algorithm can optimize the weights and biases of the BP neural network.

### 4.8. Comparison and Analysis of Prediction Effects of Different Models

Different machine learning models were used to predict the mechanical properties of the same material, with the same input variables for all models. Due to the different learning methods of different models on data, there are differences in the objective function, complexity, and hyperparameter settings of the models. Therefore, the prediction accuracy of different models varies. Table 8 compares the performance evaluation metrics of different models on the test dataset, including *R*^2^, the MAE, MRE, and RMSE. The values of *R*^2^ for the BP, PSO-BP, ELM, SVR-Poly, and SVR-RBF models on the test set were all above 0.9. In contrast, the *R*^2^ values for the SVR-Linear and RF models on the test set were 0.547 and 0.799, respectively, which were lower than those of the other models, indicating that the SVR-Linear and RF models have a weaker ability to fit the new data compared to the other models. The SVR-Linear, SVR-Poly, and RF models require a relatively short training time, and the models are trained faster. However, the MAE and RMSE evaluation index values of the three models are relatively high, and the prediction accuracy of the models is low.

All evaluation index values of the PSO-BP, SVR-RBF, and ELM models, except for the MRE value, were relatively small, with no significant differences among the three models. To further evaluate the performance of the model, paired Wilcoxon signed rank tests were performed on the absolute error values of the test samples using PSO-BP, SVR-RBF, and ELM models to determine the significance of the model. If the *p*-value is greater than 0.05, it is considered that there is a significant difference between the models; otherwise, it is considered that there is no significant difference between the models. The results of the Wilcoxon signed rank test results are shown in Table 9. As can be seen from Table 9, the *p*-values of the Wilcoxon signed rank test between the SVR-RBF, PSO-BP, and ELM models are all greater than 0.05, and there is no significant difference between the models.

**Figure 10:**
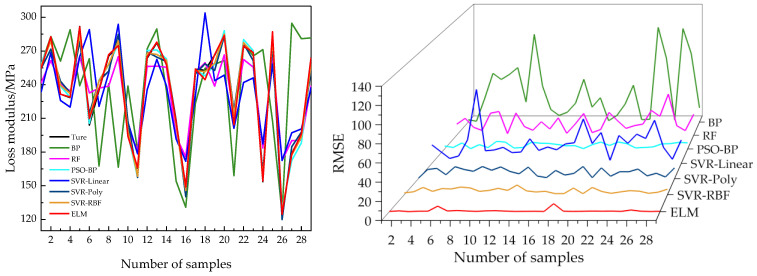
Comparison of prediction performance of different models (**left**) and error comparison chart (**right**).

Based on Table 8, the *R*^2^ values of the PSO-BP, SVR-RBF, and ELM models on the test set are close to 1, indicating that all three models have a good data-fitting ability. Among all evaluation indicators, the MAE and RMSE values of the ELM model are lower than those of the PSO-BP model and the SVR-RBF model, except for the MRE value, which is slightly higher than those of the other two models. Considering the time required for model training, the ELM model has the shortest training time, indicating that its training speed is faster than the other models’. The comparison of prediction performance and error between different models is shown in Figure 10. From the figure, it can be seen that the ELM model has better prediction performance and a more stable error fluctuation curve than other models. The data in this experiment has the characteristic of small samples, and the SVR model is suitable for processing small-sample data. However, it can be seen from the results of this experiment that the ELM model has better performance.

In summary, the ELM model introduced in this article demonstrates a better data-fitting ability and prediction accuracy, and has good prediction performance.

## 5. Conclusions

Seven different proportions of Masson pine and Chinese fir mixed-fiber-reinforced HDPE composites were prepared using the extrusion molding method, and their dynamic thermomechanical properties were tested and analyzed. As the temperature increased, the storage modulus of WPCs showed a decreasing trend. Reducing the mass ratio of Masson pine wood fibers to Chinese fir wood fibers led to an increase in the storage modulus. When the mass ratio of Masson pine wood fibers to Chinese fir wood fibers was 1:5, the storage modulus of the WPCs reached the highest value. The loss modulus of the composites increased as the content of Masson pine fiber decreased, with the lowest loss modulus was observed in the HDPE composites reinforced with Masson pine wood fibers. The loss tangent of all seven types of WPCs increased with rising temperature. The largest loss tangent was observed in the WPCs reinforced with Masson pine wood fibers and HDPE.

The sample data for the dynamic thermomechanical properties of WPCs was obtained from experimental measurements. Due to the limitations of the experimental environment and equipment, the sample size is relatively small, which limits the generalization ability of the model during the training process and makes it prone to overfitting.

In subsequent research, the prediction accuracy and generalization ability of the ELM model is expected to significantly improved if the training dataset can be expanded. This will make it more feasible to predict the dynamic thermomechanical properties and behaviors of other composites.

## Figures and Tables

**Table 1:** Mass fraction of HDPE composites reinforced with a mixture of Masson pine and Chinese fir fibers.

No.	Masson Pine Fiber/%	Chinese Fir Fiber/%	HDPE/%	MAPE and Wax/%
1	60	0	36	4
2	50	10	36	4
3	40	20	36	4
4	30	30	36	4
5	20	40	36	4
6	10	50	36	4
7	0	60	36	4

**Table 2:** Commonly used kernel functions.

Name	Representation	Parameter
Linear	$K\left(x_{i},x_{j}\right)=x_{i}^{T},x_{j}$	
Poly	$K\left(x_{i},x_{j}\right)={\left(x_{i}^{T},x_{j}\right)}^{d}$	d≥1
RBF	$K\left(x_{i},x_{j}\right)=\exp\left(-\frac{||x_{i}-x_{j}|{|}^{2}}{2\sigma^{2}}\right)$	σ>0
Laplace	$K\left(x_{i},x_{j}\right)=\exp\left(-\frac{\left||x_{i}-x_{j}|\right|}{\sigma^{2}}\right)$	σ>0
Sigmoid	$K\left(x_{i},x_{j}\right)=\tanh\left(\beta x_{i}^{T}x_{j}+\theta \right)$	β>0,θ<0

**Table 3:** Different model parameter settings.

Model	Parameter Settings
SVR	C = 4.0; g = 0.8; kernel function: Linear, Poly, RBF
RF	Number of decision trees: 500; minimum number of leaves: 6
BP	Number of neurons in the hidden layer: 15; hidden layer activation function: tansigmoid; output layer activation function: purelin; g = 10^−6^; lr = 0.01
PSO-BP	*c*_1_ = 4.494; *c*_2_ = 4.494; axgen = 15; sizepop = 10; *V*_max_ = 1.0; *V*_min_ = −1.0; popmax = 1.0; popmin = −1.0;
ELM	Number of hidden layer nodes: 50; activation function: Sigmoid

**Table 4:** Comparison of various evaluation indicators of different kernel function models on the test set.

Kernel	*R* ^2^	MAE	MRE	RMSE
SVR-Linear	0.547	22.826	0.105	29.444
SVR-Poly	0.960	7.137	0.032	8.651
SVR-RBF	0.985	4.452	0.021	5.296

**Table 5:** Comparison of evaluation indicators between ELM model training set and test set.

Set Name	*R* ^2^	MAE	MRE	RMSE
Training set	0.999	0.351	0.003	0.504
Test set	0.992	1.363	0.526	3.311

**Table 6:** Comparison of various evaluation indicators on the training and testing sets of the Random Forest model.

Set Name	*R* ^2^	MAE	MRE	RMSE
Training set	0.847	13.705	6.831	17.777
Test set	0.799	15.864	7.914	19.849

**Table 7:** Comparison of evaluation indicators between BP and PSO-BP model test sets.

Set Name	*R* ^2^	MAE	MRE	RMSE
BP	0.981	4.603	0.022	5.815
PSO-BP	0.989	3.167	0.185	4.342

**Table 8:** Comparison of prediction performance of different models on the test set.

Model Name	*R* ^2^	MAE	MRE	RMSE	Time/s
BP	0.981	4.603	0.022	5.815	21.8
PSO-BP	0.989	3.167	0.185	4.342	41.7
SVR-Linear	0.547	22.826	0.105	29.444	31.1
SVR-Poly	0.960	7.137	0.032	8.651	24.0
SVR-RBF	0.985	4.452	0.021	5.296	23.5
RF	0.799	15.864	7.914	19.849	26.7
ELM	0.992	1.363	0.526	3.311	16.8

**Table 9:** Wilcoxon signed rank test results.

Project	ELM and PSO-BP	ELM and SVR-RBF	SVR-RBF and PSO-BP
Positive rank sum	225	238	227
Negative rank sum	210	197	208
*p*-value	0.642	0.922	0.770

## Data Availability

Data is contained within this article.
